# A Semiautomated Chart Review for Assessing the Development of Radiation Pneumonitis Using Natural Language Processing: Diagnostic Accuracy and Feasibility Study

**DOI:** 10.2196/29241

**Published:** 2021-11-12

**Authors:** Jordan McKenzie, Rasika Rajapakshe, Hua Shen, Shan Rajapakshe, Angela Lin

**Affiliations:** 1 Northern Medical Program Faculty of Medicine University of British Columbia Prince George, BC Canada; 2 Medical Physics BC Cancer Kelowna, BC Canada; 3 Department of Surgery Faculty of Medicine University of British Columbia Vancouver, BC Canada; 4 Department of Mathematics and Statistics University of Calgary Calgary, AB Canada; 5 Island Medical Program Faculty of Medicine University of British Columbia Victoria, BC Canada; 6 Radiation Oncology BC Cancer Kelowna, BC Canada

**Keywords:** chart review, natural language processing, text extraction, radiation pneumonitis, lung cancer, radiation therapy, python, electronic medical record, accuracy

## Abstract

**Background:**

Health research frequently requires manual chart reviews to identify patients in a study-specific cohort and examine their clinical outcomes. Manual chart review is a labor-intensive process that requires significant time investment for clinical researchers.

**Objective:**

This study aims to evaluate the feasibility and accuracy of an assisted chart review program, using an in-house rule-based text-extraction program written in Python, to identify patients who developed radiation pneumonitis (RP) after receiving curative radiotherapy.

**Methods:**

A retrospective manual chart review was completed for patients who received curative radiotherapy for stage 2-3 lung cancer from January 1, 2013 to December 31, 2015, at British Columbia Cancer, Kelowna Centre. In the manual chart review, RP diagnosis and grading were recorded using the Common Terminology Criteria for Adverse Events version 5.0. From the charts of 50 sample patients, a total of 1413 clinical documents were obtained for review from the electronic medical record system. The text-extraction program was built using the Natural Language Toolkit Python platform (and regular expressions, also known as RegEx). Python version 3.7.2 was used to run the text-extraction program. The output of the text-extraction program was a list of the full sentences containing the key terms, document IDs, and dates from which these sentences were extracted. The results from the manual review were used as the gold standard in this study, with which the results of the text-extraction program were compared.

**Results:**

Fifty percent (25/50) of the sample patients developed grade ≥1 RP; the natural language processing program was able to ascertain 92% (23/25) of these patients (sensitivity 0.92, 95% CI 0.74-0.99; specificity 0.36, 95% CI 0.18-0.57). Furthermore, the text-extraction program was able to correctly identify all 9 patients with grade ≥2 RP, which are patients with clinically significant symptoms (sensitivity 1.0, 95% CI 0.66-1.0; specificity 0.27, 95% CI 0.14-0.43). The program was useful for distinguishing patients with RP from those without RP. The text-extraction program in this study avoided unnecessary manual review of 22% (11/50) of the sample patients, as these patients were identified as grade 0 RP and would not require further manual review in subsequent studies.

**Conclusions:**

This feasibility study showed that the text-extraction program was able to assist with the identification of patients who developed RP after curative radiotherapy. The program streamlines the manual chart review further by identifying the key sentences of interest. This work has the potential to improve future clinical research, as the text-extraction program shows promise in performing chart review in a more time-efficient manner, compared with the traditional labor-intensive manual chart review.

## Introduction

### Background

**Figure 1:**
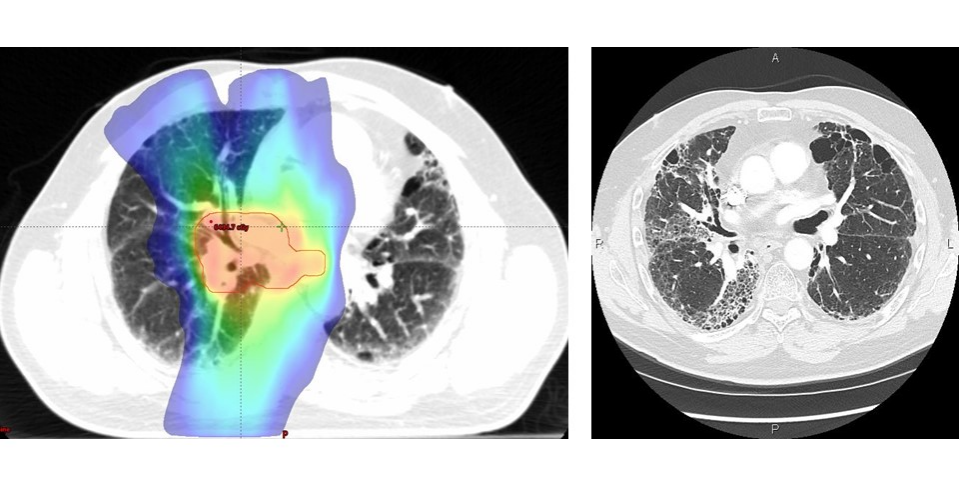
Color wash of the dose distribution on a radiation therapy planning computed tomography for a patient with lung cancer (left). The blue edge represents the 20 gray dose line, which is the recognized dose associated with increased risk of radiation pneumonitis. The same patient’s 3-month follow-up computed tomography image showed opacity indicating a partial filling of the air spaces in the lungs. These radiologic changes are representative of radiation pneumonitis in the radiation field (right).

Retrospective chart reviews require the analysis of pre-existing clinical data to answer a research question. To identify the patient cohort of interest, researchers often need to use certain inclusion criteria to scan a large *database*. After the patient cohort is identified, data abstraction begins, and a number of patient variables can be collected [1-3]. For example, cancer research frequently uses chart reviews to examine the outcomes and specific side effects of therapies. Radiation pneumonitis (RP) is a potential side effect of radiation therapy (RT) in patients with lung cancer, which can lead to permanent lung damage visible on radiography (Figure 1) [4,5]. Patients with RP may develop supplemental oxygen dependence and have a lower quality of life; as such, it is an important outcome to consider after RT and important to understand factors that may increase or decrease the likelihood of its development [4]. Of the patients with lung cancer treated with RT, it is expected that approximately 10% to 20% will develop moderate to severe RP [6-9]. Although RP fatality is uncommon, it still occurs in 1.9% of those affected [10]. For selecting a cohort of patients who developed symptomatic RP, the charts of patients with stage 2-3 lung cancer who received curative RT during the study period must be reviewed. In a typical manual chart review, this would involve researchers going through patient charts and looking for evidence and severity of RP diagnosis based on the Common Terminology Criteria for Adverse Events (CTCAE) version 5.0 [9]. This process takes significant human resources and time to identify the patient cohort of interest [11,12]. The time requirement is amplified in cohorts that have a small representation in the larger data set, where a much larger data set is necessary to be reviewed to find a significant number of rare events [12]. This decreases the chart review productivity, where a high percentage of the chart review process will be unfruitful in identifying patients for the cohort and can be seen as a loss of valuable research time. Our goal is to use a computer program developed in-house to assist in the identification of the cohort of interest and move toward an automated chart review process.

The most recent and sophisticated computer programs designed to assist in chart review studies have implemented natural language processing (NLP) [13-16]. NLP is a computer model that can manipulate a document's narrative text and speech, also known as natural language, and export it in a structured format for analysis [16]. This type of modeling is necessary because of the nature of electronic medical records (EMRs). Typically, patient charts in the EMR are written in a narrative text format, which is more difficult for a computer program to extract information from compared with a structured charting system that is arranged in tables [17]. It has been estimated that up to 80% of health care data are in an unstructured narrative format within most EMR systems [18]. Using an NLP computer algorithm as a tool could enable a chart review to be completed in less time with less human resources.

### Objective

The objective of this study is to evaluate the feasibility and accuracy of an in-house developed rule-based text-extraction program written in Python to identify patients with lung cancer who developed RP after receiving curative RT. This rule-based text-extraction program written in Python is the first stage of developing a more robust NLP program. RP is an important factor to consider with respect to RT and serves as a marker for treatment-specific variables and allows us to evaluate the use of the text-extraction program. Specifically, the focus of identification in this study is on clinically significant cases of grade ≥2 RP. RP is graded by severity; if the patient’s quality of life is affected by shortness of breath and cough, it is grade ≥2, whereas grade 1 RP is only seen on imaging and is not associated with any symptoms (Textbox 1) [9].

**Radiation pneumonitis (RP) grading based on Common Terminology Criteria for Adverse Events version 5.0.**

**Grade 0**

- No RP present

**Grade 1**

- Asymptomatic; clinical or diagnostic observations only; intervention not indicated

**Grade 2**

- Symptomatic; medical intervention indicated; limiting instrumental activities of daily living

**Grade 3**

- Severe symptoms; limiting self-care activities of daily living; oxygen indicated

**Grade 4**

- Life-threatening respiratory compromise; urgent intervention indicated (eg, tracheotomy or intubation)

**Grade 5**

- Death

## Methods

### Recruitment

The study population included a sample subset of 50 patients, from those who received curative RT for stage 2-3 non–small cell lung cancer from January 1, 2013, to December 31, 2015, at British Columbia (BC) Cancer Kelowna. The sample subset was designed to represent the proportions of RP grades in the literature [6-8,10,19]. However, there is a lack of consensus on the proportions of RP grades among patients treated with RT, most likely because of the numerous variables identified in contributing to RP development, including age, RT dose, concurrent chemotherapy, and underlying comorbidities such as chronic obstructive pulmonary disease [6-8,10,19]. The sample subset represents the proportion of RP grades most likely to be encountered in a larger randomized data set. Once the proportions of RP grade were decided on for the cohort based on the literature, simple random sampling without replacement was done on the manually reviewed cohort.

### Data Exclusion

Patients who underwent surgery after radiation treatment were excluded. Patients who received palliative radiation and patients with small cell lung cancer were excluded.

### Workflow

A manual chart review was completed by reviewing patient charts from the institutional EMR at BC Cancer Kelowna. The manual chart review results served as the definitive diagnosis, with which the assisted chart review program was compared. In the manual chart review, RP diagnosis and grading were recorded using CTCAE version 5.0 (Textbox 1) [9].

The in-house text-extraction program was built using the Natural Language Toolkit Python platform (and regular expressions, also known as RegEx). Patient charts were extracted from the BC Cancer EMR system and were subsequently formatted into the American Standard Code for Information Interchange text files to be compatible with the text-extraction program. From the charts of 50 sample patients, a total of 1413 clinical documents (clinical notes and radiology reports) were obtained for review. The reports from the BC Cancer EMR system were obtained by either direct conversion to text format documents or were printed in PDF and then converted to text format using the open-source Python Tesseract optical character recognition program. This step of obtaining and converting the documents to text format from the BC Cancer EMR system was necessary, as the text-extraction program input requires text format documents. Python version 3.7.2 was used to run the assisted chart review text-extraction program. The terms *pneumonitis*, *radiation pneumonitis*, *radiation induced lung injury*, and *fibrosis* were used as key terms for the assisted chart review. These key terms were chosen by the radiation oncologist contributing clinical expertise in this study, and they represent terminology that a physician would use to identify RP in dictated reports. The output of the text-extraction program was a list of full sentences containing the key terms, along with the document IDs and dates from which these sentences were extracted. The text-extraction program was designed to search through all the charts and extract the whole sentence that contained the key terms. If a sentence was extracted from a patient’s chart, the patient was identified as having RP. The text-extraction program organized the extracted information, identified the patients, and indicated the exact documents containing the key terms. The results from the text-extraction program were then compared with those from the manual chart review.

If the text-extraction program is shown to be feasible and accurate, a more expedited manual chart review can be performed using the results of the text-extraction program in future studies. Patients with no key terms identified in their charts will be designated as grade 0 RP, and no further chart review of these patients will need to be completed. For the patients identified by the text-extraction program to have RP, the sentences containing the key terms can be reviewed manually, first to confirm that these patients are correctly identified as having RP, and then to grade the RP severity in an expedited manner. Thus, there is an opportunity to improve the text-extraction program specificity during this sentence review process by correcting the false-positive cases.

### Statistical Analysis

The comparison between the manual chart review and text-extraction program output was viewed and analyzed in 2 different ways: the first approach considered the diseased state to be grade ≥1 RP, and the second approach considered the diseased state to be grade ≥2 RP, with grade 1 RP classified as a healthy state as the patients with grade 1 RP had no clinical symptoms. The text-extraction program was designed to look for any grade of RP when searching through the patient charts, so this lends itself to being able to perform well during the first analysis. However, grade 1 RP is only visible radiographically and thus is not clinically relevant to a patient’s further care. Thus, we wanted to look at how well the assisted chart review system was able to identify patients with symptomatic RP. Statistical analyses were performed using SAS software version 9.4.

## Results

### Text-Extraction Program Output

The results of the text-extraction program used to identify patients with RP of any grade are shown in Tables 1 and 2. The text-extraction program was able to ascertain 92% (23/25) of patients who developed grade ≥1 RP (sensitivity 0.92, 95% CI 0.74-0.99; specificity 0.36, 95% CI 0.18-0.57). The results of the text-extraction program used to identify patients with symptomatic RP, that is, grade ≥2, is shown in Table 3. The text-extraction program was able to correctly identify all 9 patients with grade ≥2 RP (sensitivity 1.0, 95% CI 0.66-1.0; specificity 0.27, 95% CI 0.14-0.43). Both analyses revealed that the text-extraction program was capable of significantly differentiating between the diseased and healthy groups.

**Table 1:** The assisted chart review text-extraction program results and the accuracy for each RP grade.

RP severity (grade)	Total, N	Correctly identified, n (%)
0	25	9 (36)
1	16	14 (88)
2	7	7 (100)
3	2	2 (100)

**Table 2:** The assisted chart review text-extraction program results for differentiating between patients with radiation pneumonitis (RP) of grade 0 (healthy) versus those with RP of grade ≥1 (diseased).

Text-extraction program findings	Manual chart review finding		
	Healthy (grade 0 RP), n (%)	Diseased (grade ≥1 RP), n (%)	Total, N
Healthy (grade 0 RP)	9 (18)	2 (4)	11
Diseased (grade ≥1 RP)	16 (32)	23 (46)	39
Total	25 (50)	25 (50)	50

**Table 3:** The assisted chart review text-extraction program results looking at the ability to distinguish between patients with radiation pneumonitis (RP) of grade ≤1 (healthy) and those with of grade ≥2 (diseased).

Text-extraction program findings	Manual chart review finding		
	Healthy (grade ≤1 RP), n (%)	Diseased (grade ≥2 RP), n (%)	Total, N
Healthy (grade ≤1 RP)	11 (22)	0 (0)	11
Diseased (grade ≥2 RP)	30 (60)	9 (18)	39
Total	41 (82)	9 (18)	50

The text-extraction program missed 2 patients with grade 1 RP. Upon further review, the 2 patients with grade 1 RP that the text-extraction program *missed* were found to truly have grade 0 RP but were incorrectly labeled as patients with RP because of human error in the manual chart review. If we correct for this human error, the sensitivity improves to 1.0 for the text-extraction program’s ability to identify grade ≥1 RP.

### Clinical Utility

In our cohort, each patient’s chart consisted of an average of 28 clinical documents that make up their chart, with a range of 15 to 150 documents. The average time spent during the manual chart review of one patient’s chart was 30 minutes. Therefore, the manual chart review of the 50-patient cohort took 25 hours. In comparison, the assisted chart review text-extraction program processed the 1413 clinical documents and exported the results in <5 minutes.

The use of the text-extraction program in this study would be to avoid unnecessary manual review of 22% (11/50) of the sample, including their electronic documents (198/1413, 14%), as these patients were identified as not having RP and thus would not require any manual review. It will also streamline the rest of the manual review as key sentences with the key terms are identified, thus further reducing the number of clinical documents necessary for the manual review to confirm that the patient should be included in the cohort.

## Discussion

### Principal Findings

The text-extraction program was able to identify patients with RP with high sensitivity but, unfortunately, low specificity. This can assist in the identification of a patient cohort of interest in a more efficient manner.

The text-extraction program correctly identified 2 patients with grade 0 RP that the manual chart review incorrectly identified. Similar findings have been reported in the literature, where one study found that their automated chart review outperformed their manual chart review as the human reviewer missed the correct classification on manual evaluation of the chart [11]. Therefore, although the gold standard for assessing the accuracy of the text-extraction program in this study is manual chart review, the process is very tedious and not guaranteed to be perfect because of human error [11,20]. This highlights a potential advantage of the text-extraction program at being more accurate than the human-led manual chart review.

The utility of the text-extraction program in this study would be to perform a rapid scan of a larger data set of documents and avoid unnecessary manual review of many of the non-RP patient charts. The program is able to use key terms, such as RP or fibrosis, to return a list of patients with those terms in the patient charts. This will significantly cut down on the number of charts that the manual review will include. This is mainly because of the fact that even if a patient does develop RP, most of their charts do not include any indication of their diagnosis. The computer program organizes the extracted information into which patient and which exact chart, thus further reducing the amount of chart review that is necessary to manually review to confirm that the patient should be included in our cohort.

The end goal of using text-extraction programs to perform chart reviews is to save the researcher time and effort of combing through patient charts to form a cohort in which to begin studying a clinical outcome. Our text-extraction program was able to output its results in <5 minutes compared with the 25 hours it took the manual chart review control to create the RP cohort.

### Limitations

A limitation to implementing this assisted chart review program is its current high false-positive rate, leading to unnecessary chart review of patients with no RP. The development of automated chart reviews must consider the balance between NLP program accuracy (no diseased cases missed) versus the amount of time saved by confidently eliminating true RP grade 0 patients in the review. Designing the key terms was an important process to balance the accuracy of the text-extraction program versus the time saved using the text-extraction program. Selecting broad key terms is important to capture all patients who may fall into our cohort; however, more specific key terms would better rule out patients not within the study cohort. Our goal was to maximize the sensitivity of the text-extraction program by including broad terms so as to not miss any patients with the diseased state initially, as the sentence output of the text-extraction program allows for a truncated chart review to improve the specificity. This means that the possible time saved in this feasibility study was less as more false-positive RP patients were identified. Future work is underway to improve the specificity of the text-extraction program with a larger sample.

Another limitation of our work is the small sample size of 50 patients. This sample group was used as a proof of concept for our in-house developed text-extraction program. This study’s results will guide further refinement of the text-extraction program and validation with a larger sample of patients.

The rule-based text-extraction program used in this study still requires human involvement in a number of steps. The clinical documents in the BC Cancer EMR system had to be obtained manually rather than automatically, which continues to pose a barrier in making chart review research as time efficient as possible.

In addition, it is important to point out that expert opinions were necessary to identify the key terms to be used in the text-extraction program. This is not only another human involvement requirement but also indicates that the results are dependent on the quality of the expert. In addition, this makes the program less generalizable to other cohorts without a new expert to create the proper key terms for each specific cohort.

### Comparison With Previous Work

Other studies have used NLP programs to assist with chart reviews in many scopes of medicine, including respirology, cardiology, and neurosurgery, and now our cancer research to identify patients who developed RP [21-24]. NLP has different applications in medical research, such as identifying patient cohorts such as our study and similar studies that identified cohorts of progressive heart failure and patients with asthma [21,22]. Other studies have used NLP programs to extract specific clinical features from clinical charts, such as tuberculosis patient factors and radiology characteristics of glioblastoma [23,24]. Our use of an NLP program to extract information based on key terms to reduce the amount of chart review necessary is similar to the study by Cao et al [25], where they used search terms to identify medical errors through patient charts. This allowed their group to reduce the number of charts that needed to be reviewed, from 286,000 discharge summaries to 2744 discharge summaries that were found to contain the search terms [25]. This meant that the Cao et al [25] manual review only had to be done on <1% of the initial data set. Reducing the number of charts to review saves many hours of manual chart review and would greatly increase the speed at which the review could be completed. Thus, an assisted chart review program opens the possibility of expanding the study, including a much larger data set that would be impractical to review manually. Our study adds to the existing literature on this topic by supporting the validity of NLP programs; it demonstrates the ability to further analyze an identified patient cohort based on variables of interest, such as illness severity.

### Conclusions

In conclusion, the NLP-based text-extraction program used in this study is a feasible and valuable method for identifying patients who developed RP after curative radiotherapy. First, the text-extraction program helped save chart review time by completely eliminating patient charts identified with grade 0 RP. Second, the text-extraction program extracted key sentences from patient charts and allowed for an efficient review of relevant phrases, should this be needed to grade patients’ RP severity without having to peruse the rest of their charts. For example, in a quick scan, a researcher would be able to read only the sentences with the identified keyword in a patient’s chart instead of sifting through many full documents.

The analysis revealed that the text-extraction program was capable of significantly differentiating between diseased and healthy groups. Compared with the manual chart review of the 50-patient cohort that took 25 hours, the text-extraction program was able to process all the charts in <5 minutes and exported the list of patients that had RP mentioned somewhere in their chart.

This work has the potential to improve future clinical research as the text-extraction program shows promise in performing chart review in a more time- and effort-efficient manner compared with the traditional manual chart review. The text-extraction program is available by contacting the authors (RR).

## Abbreviations

BC: British Columbia
CTCAE: Common Terminology Criteria for Adverse Events
EMR: electronic medical record
NLP: natural language processing
RP: radiation pneumonitis
RT: radiation therapy
